# The Usefulness of Evenness

**DOI:** 10.1111/ele.70446

**Published:** 2026-08-16

**Authors:** Ryan A. Chisholm

**Affiliations:** ^1^ Department of Biological Sciences National University of Singapore Singapore Singapore

**Keywords:** dominance, evenness, immigration, inequality, Lorenz consistency, productivity

## Abstract

Evenness measures similarity in abundance across species. The term ‘evenness’ was introduced to ecology in 1964, but debate persists about precisely how to measure it and its usefulness as a concept. Here I provide a perspective on both matters. For how to choose evenness metrics, I echo previous calls for the application of the Lorenz‐consistency axioms. These axioms are largely non‐controversial but have often been neglected, leading to some ongoing use of evenness indices with unintuitive properties. Two widely used evenness metrics, Hill evenness and mean pairwise similarity, satisfy the axioms and are recommended. To assess the usefulness of evenness in ecological research, I focus on two case studies. The first explores how evenness can drive community biomass. The second explores how evenness can indicate the strength of immigration into an ecological community. I conclude that, with appropriately chosen metrics, the concept of evenness can facilitate productive research programmes. Therefore, despite some recently raised objections to it, evenness is a useful concept in ecology.

## Graphical abstract

Is the concept of evenness useful in ecology? I argue that it is, because it can shed light on the mechanisms that structure ecological communities. The two graphs above illustrate how in synthetic communities evenness is related to community biomass (left) and to the immigration rate (right).

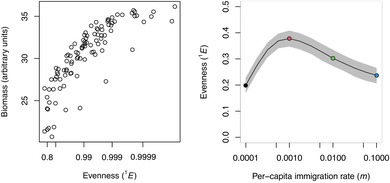

## 1 Introduction

The term ‘evenness’ in ecology goes back to Lloyd and Ghelardi (1964), and has since become used widely (Tuomisto 2012), including in undergraduate textbooks (e.g., Molles Jr. 2015). The concept of evenness is intuitive: a community in which all species have similar abundance is even, while a community with some very rare and some very common species is uneven. Nailing down a precise definition is harder. It is clear that a community with abundances $\left\{10,10,10\right\}$ is more even than the community $\left\{1,10,100\right\}$. But is the community $\left\{1,1,10\right\}$ more or less even than the community $\left\{1,10,10\right\}$?

The motivation for evenness indices arises from the need to summarise species abundance distributions (SADs), beyond just measuring species richness (May 1975). Evenness indices might be unnecessary if we had a universal model of SADs that provided a good fit to all empirical datasets—in that case we could just use the fitted model's parameters as our summary indices and compare them across communities (Pielou 1977). But, although some empirical SADs are well described by distributions such as the lognormal (Preston 1948) and the log‐series (Fisher et al. 1943), a general model that can fit all empirical SADs is still lacking (Ulrich et al. 2010). The two‐parameter combination of evenness and richness provides a way of comparing the diversity of ecological communities regardless of the precise functional forms of their SADs (May 1975; Pielou 1977).

The initial development of evenness indices focussed on factoring out species richness from diversity indices (Lloyd and Ghelardi 1964; Pielou 1966; Sheldon 1969). This led to the creation of indices such as the Pielou index, which is the Shannon index divided by its maximum value given species richness (Pielou 1966). Later, some of these indices were discovered to have undesirable properties, for example, the Pielou index always approaches unity as the number of species increases (Alatalo 1981). These problems arise from the indices' lack of an axiomatic background (Gosselin 2006). Axiomatising the evenness problem involves first stating a list of requirements for an evenness index and then using these to test existing indices or develop new ones (Engen 1979; Taillie 1979; Smith and Wilson 1996). A notable axiomisation came with the importation of the Lorenz‐consistency axioms from economics, where they undergird metrics of wealth and income inequality (Taillie 1979). The two most important Lorenz‐consistency axioms are that a transfer of abundance from a common to a rare species should increase evenness (in economics, this has been called the Robin Hood principle, because it involves taking money from the rich and giving it to the poor; Arnold 1987) and that replicating a community by doubling the number of species and repeating the abundances should not change evenness. The Lorenz‐consistency axioms, which will be explained in more detail below, have subsequently achieved support among ecologists (Gosselin 2001; Tuomisto 2012), although some dissent remains (Jost 2010). One widely used evenness index, the ratio of the exponential of the Shannon index (the Hill number of order 1; Hill 1973) to species richness, is Lorenz‐consistent; most others are not.

However, the concept of evenness is not without its problems. The multiplicity of evenness indices and the continued use of indices outside of an axiomatic framework pose challenges for interpretation of evenness values and comparisons across studies. It is worth pausing to evaluate the continued relevance of evenness in ecology (Alroy 2025). No amount of mathematical elegance or theoretical musing can alone justify a scientific concept. Concepts should be evaluated by how useful they are, which means the degree to which they further scientific progress. Pielou (1977) herself recognised that ‘Indices should be calculated for the light (not the shadow) they cast on genuine ecological problems’.

My main goal in this paper is to evaluate, over half a century since the advent of evenness indices, the usefulness of evenness in ecology. As a preamble, I review the Lorenz‐consistency axioms (Taillie 1979; Gosselin 2001; Tuomisto 2012) and give my perspective on alternative axiomatisations of the evenness problem (e.g., Jost 2010). I then explore two case studies of how evenness can further ecological research. In light of the case studies, I argue that the concept of evenness is still useful in ecology. I nevertheless discuss some of the potential limitations of evenness, in the context of a recent critique (Alroy 2025).

## 2 Lorenz Consistency

A useful evenness index should reduce a vector of abundances to just a single number (i.e., a scalar). Although I will refer to abundances as counts of individuals, all of my points apply to other measures of abundance, such as biomass or cover, as well. A rigorous approach to developing such indices begins by stating axioms that any sensible evenness index should satisfy. Ecologists have previously drawn on the concept of Lorenz consistency (Taillie 1979; Gosselin 2001; Tuomisto 2012) from economics (Arnold 1987). An evenness index is Lorenz‐consistent if and only if it satisfies four axioms (Foster 1985), here expressed in ecological terms by replacing ‘individual’ with ‘species’, and ‘wealth’ or ‘income’ with ‘abundance’:

- Symmetry (anonymity): The value of an evenness index should be independent of the ordering of elements in the abundance vector.
- Replication invariance: If a community is replicated by repeating each species' abundance a fixed number of times, resulting in more species but the same relative abundance distribution, then the value of an evenness index should remain unchanged.
- Scale invariance: The value of an evenness index should be independent of the units in which abundance is measured. This is equivalent to saying that evenness indices should depend only on relative abundances, not absolute abundances.
- The Pigou–Dalton transfer principle: If abundance is transferred from a commoner species to a rarer species (a progressive transfer), then the value of an evenness index should increase (the Robin Hood principle; Arnold 1987).

In addition, evenness indices are assumed to be continuous functions of abundance for a fixed‐length vector, and the abundances are usually assumed to be positive because of the impossibility of observing species with zero abundance (unlike in economics where individuals with zero wealth are observable; Jost 2010).

**FIGURE 1:**
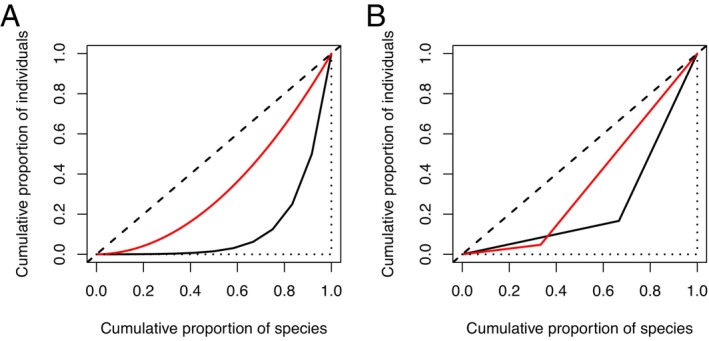
Lorenz curves, which plot cumulative proportion of individuals versus cumulative proportion of species (sorted in order of increasing abundance), allow graphical comparison of evenness across communities. (A) One community's Lorenz curve dominates that of another if the first is greater than the second at some points and never less than it. Here the red Lorenz curve, corresponding to the community $\left\{15,30,45,60,75,\ldots 1350\right\}$, with each value repeated twice, dominates the black Lorenz curve, corresponding to the community $\left\{2,4,8,16,32,\ldots 4096\right\}$, with each value repeated 15 times (these two communities have the same total abundance and same number of species). This implies that any evenness index satisfying the Lorenz‐consistency axioms will report greater evenness for the first community. The dashed line is the one‐to‐one line corresponding to the case of maximum evenness; the dotted lines show the limiting case of minimum evenness. (B) If two communities' Lorenz curves cross, then the Lorenz order is undefined. Different Lorenz‐consistent indices may thus rank the communities differently in terms of evenness. Here the red curve is for the community $\left\{1,1,10\right\}$ and the black curve is for the community $\left\{1,10,10\right\}$. Note that Lorenz curves are always constructed from discrete data via interpolation (rather than using step functions).

Lorenz consistency can be understood graphically. To construct the Lorenz curve for a community, one sorts the species by increasing abundance and then plots the cumulative fraction of individuals versus the cumulative fraction of species, resulting in a curve that is necessarily monotonically increasing from zero to one. If all species have equal abundance, the Lorenz curve will be a one‐to‐one line indicating maximum evenness (Figure 1A). To the extent that species vary in abundance, the Lorenz curve will be depressed below the one‐to‐one line (Figure 1A). If one community's Lorenz curve is greater than that of another at some points and never less than it, then we say that the first Lorenz‐dominates the second (Figure 1A) or that it comes before it in the Lorenz order (Arnold 1987) and thus has greater evenness.

A pleasing feature of the Lorenz‐consistency axioms is that for some common SADs, such as the lognormal distribution and the gamma distribution, the axioms define a strict ordering of communities by evenness (Taillie 1979). More generally, however, the Lorenz‐consistency axioms on their own do not fully determine ordering. For our earlier example with the two communities with abundance vectors $\left\{1,1,10\right\}$ and $\left\{1,10,10\right\}$, different Lorenz‐consistent indices may lead to different conclusions about which community is more even, as indicated by the fact that their Lorenz curves cross (Figure 1B).

One set of Lorenz‐consistent indices is Hill evenness (Tuomisto 2012):

(1) Eq=DqS

where S is species richness and Dq is the Hill number of order q (Hill 1973; Jost 2006), which for q>1 is

Dq=∑i=1Spiq11−q

where $p_{i}$ is the relative abundance of species i, and for q=1 is equal to the exponential of the Shannon index:
(2)

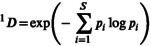




For a lognormal SAD with log‐mean μ and log‐standard deviation σ, the index E1 approaches $\exp\left(-\sigma^{2}/2\right)$ as the number of species becomes large (Appendix S1). The independence of this quantity from μ illustrates the earlier point about the Lorenz‐consistency axioms providing a unique ordering for certain well‐known SADs. Tuomisto (2012) argues that only indices like E1, which have the form diversity divided by richness, should properly be called ‘evenness’, and that the term ‘equitability’ should be used for a broader class of indices. While I find this argument plausible, I do not adhere closely to the strict terminology here. The terms ‘evenness’ and ‘equitability’ have long been used interchangeably in ecology (e.g., Taillie 1979).

Another Lorenz‐consistent evenness index is mean pairwise similarity in abundance, which is equal to one minus the Gini coefficient G, an inequality measure in economics (not to be confused with the Gini–Simpson index, which is not Lorenz‐consistent; Tuomisto 2012):

(3) $$\begin{array}{c} 1-G=1-\frac{1}{S}\sum_{i=1}^{S-1}\sum_{j=i+1}^{S}\left|p_{i}-p_{j}\right| \end{array}$$

The ecological literature on ‘dominance’, which is the flipside of evenness (Hillebrand et al. 2008), is also a source of possible indices. One way to measure dominance is $D_{u}=1-P_{u}$, where $P_{u}$ is the minimum fraction of species (i.e., the most abundant ones) accounting for an overall fraction u of individuals (ter Steege et al. 2013; Fung et al. 2015). The evenness index $P_{u}$ corresponds to a single point $\left\{u,P_{u}\right\}$ on a Lorenz curve (see Figure 1) and it satisfies a weaker version of the Lorenz‐consistency axioms where the transfer principle is modified to state that progressive transfers should not decrease evenness (rather than ‘should increase evenness’). Most other evenness indices used by ecologists are not Lorenz‐consistent (Tuomisto 2012). Economists use a variety of other Lorenz‐consistent indices, such as the Atkinson index (Atkinson 1970), that may provide future inspiration for ecologists.

One can try to improve upon the Lorenz‐consistency axioms by adding new axioms, but this usually results in redundant, contradictory, or controversial axioms. For example, one proposed additional axiom is that evenness should decrease when a very rare species is added (Tuomisto 2012), but, as I show in Appendix S2, this is actually a consequence of axioms (i–iv). Kvålseth (2015) proposed that an evenness index should be linearly related to the Euclidean distance between an abundance vector and a perfect‐evenness vector of the same length, but it is unclear why this very stringent criterion should be elevated to an axiom.

Another potential axiom comes from the proposition that the range of an evenness index should not be constrained by the value of species richness (Heip 1974; Engen 1979; Smith and Wilson 1996; Jost 2010), that is, that the minimum and maximum evenness values should be attainable for any number of species. This requirement has been referred to as ‘independence’ of evenness and richness (Jost 2010), but confusingly it is not statistical independence, which cannot be a general property of any evenness index (e.g., a log‐series SAD is governed by only one parameter that determines both richness and evenness given a fixed community size; Pielou 1977), and even more confusingly ‘independence’ has also been used to refer to replication invariance (Smith and Wilson 1996). It would be better to avoid the term ‘independence’ in future discussions of evenness axioms. I will instead refer to the requirement about the minimum and maximum values as the value‐constraint requirement. The value‐constraint requirement—specifically the constraint on the minimum value—is incompatible with the Lorenz‐consistency axioms, as can be shown graphically (Gosselin 2001) or mathematically (Appendix S3). A further objection to the value‐constraint requirement is intuition: I would argue that it is acceptable, and even desirable, for a community with more species to be potentially less even, for example, the community $\left\{1,1,1000\right\}$ seems substantially less even than the community $\left\{1,1000\right\}$.

If we were willing to discard one or more of the Lorenz‐consistency axioms, then it would be possible to create alternative axiomatisations by admitting new axioms like the value‐constraint requirement (Gosselin 2001; Gosselin 2006). For example, if we discard axiom (ii), replication invariance, and admit the value‐constraint requirement, then we obtain a set of axioms that are not satisfied by any of the above indices but are satisfied by the Pielou index J′=logD1/logS (Pielou 1966; Jost 2010) and the index D1−1/S−1 (Heip 1974). It should be mentioned that the value‐constraint requirement was apparently not actually part of Pielou's (1966) motivation for developing her index. Practitioners deciding between these two sets of axioms can ask themselves whether the evenness of the community $\left\{1,1000\right\}$ should be equal to that of the community $\left\{1,1,1000,1000\right\}$, satisfying the Lorenz‐consistency axioms, or (approximately) equal to that of the community $\left\{1,1,1000\right\}$, satisfying the alternative axioms just outlined. However, those choosing the alternative axioms should be warned that violation of replication invariance can lead to unintuitive outcomes, for example, if we take any community and replicate it large number of times, Pielou's index approaches the maximum value of 1 (Alatalo 1981) (this is straightforward to see mathematically: for a community replicated k times, giving kS species, we have $J^{\prime }=-k\sum_{i=1}^{S}\left(p_{i}/k\right)\left(\log p_{i}-\log k\right)/\left(\log k+\log S\right)$, which approaches $\sum_{i=1}^{S}p_{i}=1$ as k becomes large). For the remainder of this paper, I will take the Lorenz‐consistency axioms as given and turn to applications.

## 3 Case Study 1: Evenness and Biomass

Early research into the relationship between diversity and ecosystem function focussed mainly on species richness (Tilman 1999; Loreau et al. 2001), but the realisation that dominant species contribute to function more than rare ones has led to an increased focus on evenness (Nijs and Roy 2000; Wilsey and Polley 2004; Hillebrand et al. 2008). We may hypothesise that if a community is more even then functional‐trait diversity will be higher, leading to more effective resource exploitation and higher productivity (Zhang et al. 2012).

I illustrate the potential effects of evenness on ecosystem biomass here using the Monod consumer–resource model, which is formulated as a set of n+m differential equations, one for each of n species and one for each of m resources (Tilman 1981). The model has three parameters describing the resource uptake function for each species on each resource, a parameter for each resource's supply rate, and a parameter for the loss rate of both resources and biomass, for a total of 3mn+m+1 parameters. Equilibrium biomass in this model tends to increase with species richness (up to a maximum of n=m species) (Tilman et al. 1997; Chisholm 2025), both because of the higher chances of obtaining better‐performing species with a larger species pool (selection effects; Tilman et al. 2014) and because different species can specialise on different resources, and thus a greater number of species can more effectively exploit the resources as a whole (complementarity effects; Tilman et al. 2014).

**FIGURE 2:**
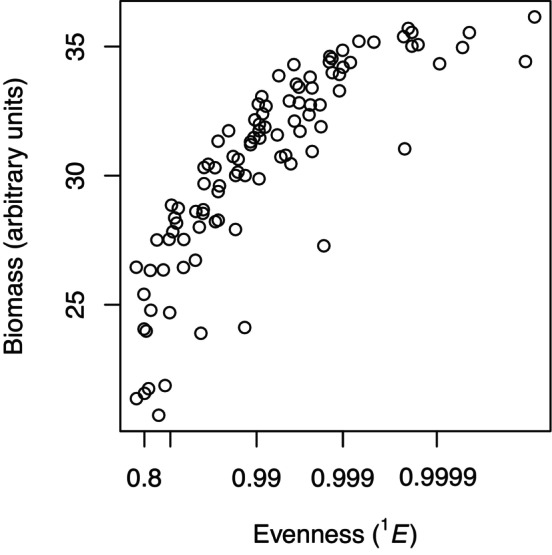
Biomass is strongly related to evenness in the Monod consumer–resource model (for the parameter values used here, $R^{2}=0.68$ for biomass and Hill evenness E1, Equations (1) and (2); note the logarithmic evenness scale on the graph). Each point represents biomass and evenness at equilibrium in one of 100 independent simulations. The model contains n=4 species and m=4 resources, and model parameters are randomly varied across simulations (see Appendix S4). In all but one simulation, all four species persisted at equilibrium.

To explore how evenness affects biomass in this model, I parameterised it with n=4 species and m=4 resources in a way that allows each species to specialise on a different resource, which usually guarantees coexistence of all species at equilibrium (Appendix S4). This allowed me to explore the influence of evenness independent of richness. I introduced a degree of randomness to the parameter values to generate variation across model runs. I then ran the model to equilibrium 100 times and recorded biomass and evenness at the end of each run. The relationship between evenness and biomass was strongly positive (Figure 2). The Hill evenness index E1 (Equations (1) and (2)) explained 68% of the variation in biomass. The abundance of the most common species explained only 10% of the variation in biomass.

Evenness is associated with high biomass in this model because if the species have similar abundances it suggests that each has effectively exploited its favoured resource, whereas if some species have low abundance then the resources on which they specialise may have been underexploited. Of course, evenness and biomass are both ultimately functions of the model parameters, and it should be possible, through a combination of careful analytical and numerical work, to obtain a more detailed mechanistic understanding here. But ‘evenness’ is a convenient one‐parameter summary statistic that gives some mechanistic insight, possibly extending beyond the details of the particular model under study, and can illuminate the path to a more detailed understanding.

Empirical evidence generally confirms the expectation that evenness should increase ecosystem function, although the results are not unanimous (Hillebrand et al. 2008). In grasslands, one early experimental study found no effect of evenness on biomass independent of species richness (Wilsey and Polley 2004), but a larger study across 28 European grasslands did find a positive and consistent effect (Kirwan et al. 2007). For forest communities, a meta‐analysis of 54 studies also found that evenness was a consistent and strong predictor of productivity—stronger than richness (Zhang et al. 2012). Evenness can also promote other ecosystem functions, such as pest control (Crowder et al. 2010). Some caution may be warranted when interpreting these results, because many studies have used evenness indices that violate Lorenz consistency. Zhang et al. (2012) and Crowder et al. (2010) used Pielou's J, which increases systematically with species richness (as noted earlier; Alatalo 1981). This does not affect the interpretation of experimental studies where richness is controlled and comparisons are made only across treatments with the same richness, but it could introduce hidden richness effects when comparing across experimental treatments or observational plots with different richness.

## 4 Case Study 2: Immigration and Evenness

My second case study concerns the use of evenness for understanding how immigration affects community structure. Some ecological communities, such as those on small islands (Lomolino 1990), are naturally subject to much lower immigration than others. Human impacts can also affect immigration, for example, by fragmenting habitat (Puettker et al. 2011). Theory predicts that immigration will usually increase community evenness, because it leads to a rescue effect that prevents species from becoming too rare (Hanski 1994).

This can be illustrated with a simple spatially implicit neutral model where immigrants to a local community come from a much larger metacommunity (Hubbell 2001; Volkov et al. 2003). In each timestep of the model, a death occurs at random in the local community and is replaced by a birth from the local community with probability 1−m or an immigrant from the metacommunity with probability m. At dynamic equilibrium, the diversity in the local community is determined by the balance between stochastic extinction due to drift and species influx due to immigration. I parameterised this model with a metacommunity of size $J_{M}=10^{8}$ and a local community size of $J=10^{6}$. I varied the per‐capita immigration rate from $m=10^{-4}$ to m=0.9. For each distinct value of the immigration rate, I set the fundamental biodiversity number θ, which governs species richness in the metacommunity, to a value that would give S=900 species in the local community at equilibrium, allowing me to explore the impact of immigration on the shape of the SAD independent of richness (see Appendix S5 for details).

**FIGURE 3:**
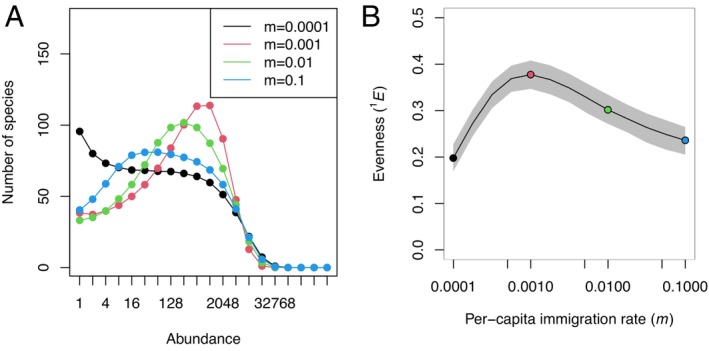
The relationship between evenness in a local community (measured here as Hill evenness, E1, Equations (1) and (2)), and the per‐capita immigration rate into the community (m) can be unimodal, as illustrated here with a spatially implicit neutral model. The model was parameterised for an expected local community size $J=10^{6}$ and expected local species richness S=900; only the immigration rate was varied (metacommunity diversity is varied concomitantly to guarantee S=900; see Appendix S5). (A) Under low immigration (black; m=0.0001), the community in this model is highly uneven (as indicated by the relatively flat SAD) because the same species rarely immigrates twice and hence immigration is unable to effectively rescue species from low abundance; under moderate immigration (red; m=0.001), the community is much more even (as indicated by the hump‐shaped SAD) because repeated immigration by the same species provides a rescue effect; under high immigration (blue and green; m=0.01 and m=0.1), the community becomes less even again because the relative abundance distribution begins to approximate that of the metacommunity, which in this model is a highly uneven log‐series distribution. The SADs are binned into $\log_{2}$ abundance categories; each plotted distribution is the mean of 1000 independent realisations of the stochastic model at dynamic equilibrium. (B) The overall relationship of evenness to per‐capita immigration rate in the model is unimodal, with a maximum near m=0.001 (black curve indicates mean relationship over 1000 realisations; grey shaded area indicates 95% prediction interval; coloured points correspond to specific values of m from panel (A)).

When immigration is very low (m=0.0001), the SAD is quite broad (Figure 3A), indicating high variation in abundance and thus low evenness (Figure 3B). As immigration increases (m=0.001), the SAD becomes more hump‐shaped (Figure 3A), indicating lower variation in abundance and thus higher evenness (Figure 3B). Perhaps counterintuitively, for even larger immigration, evenness can actually decrease again (Figure 3A,B) because the local SAD begins to resemble the metacommunity community SAD, which in this model is the log‐series—a very uneven distribution. Although this model is neutral, many of its predictions are robust to the introduction of some niche structure (Chisholm and Pacala 2010; Chisholm and Fung 2021). Its qualitative predictions about the impact of immigration on local community evenness are likely to be robust and apply to any stochastic system that experiences immigration from an uneven metacommunity.

Testing theoretical predictions about the effect of immigration on evenness with empirical data is not trivial, because of the need to control for community type, area and other covariates. One study that meets these requirements looked at arthropod communities in coastal grasslands in Portugal and found that although richness was similar on an island and the mainland, evenness was (slightly) higher on the mainland (Calado et al. 2024). More careful comparisons of this kind are needed to test our understanding of how immigration affects community structure. Controlling for species richness is essential in any such comparisons, and though in some pairwise comparisons this may be achieved fortuitously (e.g., Calado et al. 2024), in general it will require larger datasets comprising many communities.

## 5 Discussion

In a future golden age of ecology, we may have accurate mechanistic models that can be fit to data from any system of interest to facilitate prediction and understanding (Alroy 2025). Ecologists should continue to strive towards this goal. In the meantime, we will continue to rely to some degree on inferences from phenomenological summary statistics, such as evenness indices (Pielou 1977). And even in cases where appropriate mechanistic models are available, evenness may still serve as a useful heuristic for communicating complex ideas. The Lorenz‐consistency axioms eliminate much of the verbal ambiguity in the evenness concept. There remains some grey area in terms of how to order communities by evenness (Figure 1B), but there are many useful concepts in biology with grey areas, such as the species concept (Hey 2006) and niche overlap (Alatalo 1981).

My two case studies illustrate the usefulness of evenness. One could argue that the ideas that evenness increases community biomass and that evenness peaks at intermediate immigration could be reframed without any ‘evenness’ concept at all. The latter idea can be understood just by looking at the SADs (Figure 3A), but evenness provides a clearer one‐dimensional summary of the phenomenon (Figure 3B) and enables more succinct verbal communication. The need for summary statistics like evenness is even greater in empirical situations where, unlike in my synthetic case studies, we usually lack an accurate mechanistic model of the system to facilitate detailed understanding. The mechanistic model used in the evenness–biomass case study can accurately describe two‐species communities (Tilman 1981), but it is hard to parameterise for larger communities. Similarly, the mechanistic model used in the immigration–evenness case study provides good fits to some empirical SADs (e.g., Volkov et al. 2003), but not others (Ulrich et al. 2010).

Despite its usefulness, the concept of evenness does have limitations. Foremost among these is the impossibility of accurately estimating community evenness from a sample (Pielou 1977; Jost 2010; Chao et al. 2020). The problem is that rare species are inevitably underrepresented in samples (Preston 1948; Chisholm 2007), and so sample evenness usually overestimates community evenness (Appendix S6). The only way around this would be to make parametric assumptions about the form of the SAD, but such extrapolation is precarious without a solid mechanistic understanding of the system, which as already noted is often lacking. Sampling also introduces noise into SADs and thus in evenness indices, which can obscure the underlying relationships between the indices and SAD parameters discussed earlier (Alroy 2025). The recommended approach when applying evenness indices to a sample is to treat evenness as a property of the sample itself, rather than as an estimate of community evenness. To compare evenness across samples of different communities one can use coverage‐based estimators (Jost 2010; Chao et al. 2020), which are well developed for Hill numbers (Roswell et al. 2021). This facilitates testing of, for example, hypotheses about how evenness changes with environmental variables or across land‐use treatments. The above discussion assumes that sampling of communities occurs at the individual level. There have been some claims that evenness indices should instead be robust to species‐level sampling (Engen 1979; Alroy 2025), but this is not the way communities are sampled in the field and, furthermore, a useful evenness index should in fact be sensitive to the addition or removal of a single very abundant species.

Another question surrounding evenness is its relevance to conservation. It is unclear whether we should prefer ecosystems to be more even or less even (Alroy 2025). Some systems may be naturally uneven, for example, the Mora forests of Trinidad, where, atypically for a tropical forest, the species *Mora excelsa* constitutes 85%–95% of canopy trees (Beard 1946). No conservation scientist would advocate managing such a naturally low‐evenness system for greater evenness. Similarly, whereas richness is used for conservation prioritisation across space (Myers et al. 2000), there would appear to be no firm basis for prioritising areas of higher (or lower) evenness. Evenness may be most useful in conservation as a crude one‐dimensional indicator of environmental change, particularly in the early stages (Engen 1979) when species may shift in abundance without actually going extinct (Hillebrand et al. 2008). In line with my earlier comments about basic science, evenness may be a blunt tool but nevertheless a useful one for ecosystem management, in the absence of detailed mechanistic understanding.

In conclusion, while the concept of evenness has limitations, it is a broadly intuitive concept that can be placed on a firm theoretical foundation via an axiomatic approach. The crucial test for whether a concept is useful is whether it contributes to a productive research programme that improves our understanding of nature. Similar points have been made in the context of debates about other concepts in biology, including inclusive fitness (Nowak et al. 2010; Abbot et al. 2011) and niche construction (Scott‐Hillips et al. 2014). Alroy (2025) worries that evenness is not ‘real’, but it is unclear what definition of ‘real’ he is applying—puzzlingly, he believes that temperature or the σ parameter of a lognormal distribution can be real in a way that an evenness index cannot. My two case studies suggest that evenness is contributing to fruitful research programmes in ecology. Similar advances could perhaps have been made without any explicit ‘evenness’ concept. But would they have been? It is difficult to answer this counterfactual. There is a least a prima facie case that evenness has served a useful role.

## Author Contributions

R.A.C. performed modelling work and wrote the paper.

## Funding

This work was supported by Singapore's Ministry of Education (Grant WBS A‐800104600‐00).

## Supporting information


**Appendix S1:** Hill evenness applied to common abundance distributions.
**Appendix S2:** The Lorenz‐consistency axioms and addition of rare species.
**Appendix S3:** The Lorenz‐consistency axioms and Jost's evenness property.
**Appendix S4:** Evenness and biomass.
**Appendix S5:** Immigration and evenness.
**Appendix S6:** Robustness of evenness indices to sampling.
**Figure S6:1** Community evenness tends to be overestimated from samples, although estimates are somewhat robust down to a sample size of ≈50%, here with the exception of the case where the SAD is bimodal. Grey points show evenness estimates from individual samples and red curves show average evenness values for each sample size. Each panel is for a distinct SAD (panel titles). These simulations are for communities with S=10 species and an expected community size of N=1,000.
**Figure S6:2** As for Figure S6.1, but with S=100 and N=10,000.

## Data Availability

The author has nothing to report.
