# Supplementary material for: The Usefulness of Evenness
Source: Ecol Lett. 2026 Aug 16;29(8):e70446. doi: 10.1111/ele.70446 (PMC13477743; doi:10.1111/ele.70446)
Supplement: Supplementary file 1 [file ELE-29-0-s001.docx]

**Appendix S1: Hill evenness applied to common abundance distributions**

In this appendix I derive some asymptotic results demonstrating that the Hill evenness index defines a unique ordering of communities for some common SADs (Taillie 1979).

Suppose that we have a lognormal SAD with log-mean $\mu$ and log-standard deviation $\sigma$. Let abundance be the random variable $X$. The probability density function for the abundances is

$$\phi\left( x \right)=\Pr\left( X=x \right)=\frac{1}{x\sigma\sqrt{2\pi}}\exp\left( -\frac{\left( \log x-\mu\right)^{2}}{2\sigma^{2}} \right)$$

Let the number of species be $S$ and the total number of individuals (the community size) be $N$. The expected average abundance is

$$\begin{aligned} E\left( \frac{N}{S} \right)=\int_{x=0}^{\infty} \phi\left( x \right)xdx=\exp\left( \mu+\frac{\sigma^{2}}{2} \right)\#\left( A3.1 \right) \end{aligned}$$

Let $p_{i}$ be the abundance of species $i$. We can calculate the expected value of the Shannon index as follows:

$$E\left( \log{}^{1}D \right)=E\left( H \right)=E\left( -\sum_{i=1}^{S} p_{i}\log p_{i} \right)$$

Asymptotically, as the number of species becomes large, the realised relative SAD will approach the expected distribution, i.e., the density of species with relative abundance $p_{i}=x/N$ approaches $\phi(x)$, and we can write

$$E\left( H \right)=E\left( -\sum_{i=1}^{S} p_{i}\log p_{i} \right)\sim-S\int_{x=0}^{\infty} \phi(x)\frac{x}{N}\log\frac{x}{N}dx=\frac{S}{N}\int_{x=0}^{\infty} \phi(x)x\left( \log N-\log x \right)dx=\frac{S}{N}\left( \log N-\mu-\sigma^{2} \right)\exp\left( \mu+\frac{\sigma^{2}}{2} \right)$$

In the same asymptotic limit, the average abundance $N/S$ approaches its expected value from Eq. (A3.1), and we have

$$E\left( H \right)\sim\frac{S}{N}\left( \log S+\mu+\frac{\sigma^{2}}{2}-\mu-\sigma^{2} \right)\frac{N}{S}=\log S-\frac{\sigma^{2}}{2}$$

May (1975, Eq. (A.18)) presents a much more complicated formula for the expected value of the Shannon index for a lognormal SAD, the difference apparently mainly arising because he truncates the distribution at fixed minimum and maximum abundances. The extra analytical complexity introduced by truncation is hard to justify: although a truncated lognormal is arguably more biologically realistic, it is still a highly idealised representation of a real community.

For a lognormal SAD, Pielou’s index for given $S$ is

$$E\left( J \right)=\frac{\log{}^{1}D}{\log S}\sim\frac{1}{\log S}\left( \log S-\frac{\sigma^{2}}{2} \right)=1-\frac{\sigma^{2}}{2\log S}$$

This shows that (asymptotically) the expected value of Pielou’s index for a lognormal SAD depends only on $\sigma$, not $\mu$. It also confirms the pathological behaviour of the index, mentioned in the main text, whereby $E\left( J \right)\to1$ as $S\to\infty$.

For the Hill index of order 1, for a given $S$, we have

$$E\left( {}^{1}E \right)=E\left( \frac{{}^{1}D}{S} \right)$$

As the number of species becomes large ($S\to\infty$), we have $\log{}^{1}D\sim E\left( \log{}^{1}D \right)=\log S-\frac{\sigma^{2}}{2}$, and thus ${}^{1}D\sim S\exp\left( -\frac{\sigma^{2}}{2} \right)$ and

$${}^{1}E=\frac{{}^{1}D}{S}\to e^{-\frac{\sigma^{2}}{2}}$$

Again this is independent of $\mu$ (and $S$).

Now consider the gamma SAD with parameters $\alpha$ and $\theta$:

$$\phi\left( x \right)=\frac{1}{\Gamma\left( \alpha\right)\theta^{\alpha}}x^{\alpha-1}e^{-\frac{x}{\theta}}$$

The expected average abundance is

$$\begin{aligned} E\left( \frac{N}{S} \right)=\int_{x=0}^{\infty} \phi\left( x \right)xdx=\alpha\theta\#\left( A3.2 \right) \end{aligned}$$

Following similar logic to that above, we find that the expected value of the Shannon index as the number of species becomes large is

$$E\left( \log{}^{1}D \right)=E\left( H \right)\sim-\int_{x=0}^{\infty} S\phi(x)\frac{x}{N}\log\frac{x}{N}dx=-\int_{x=0}^{\infty} S\frac{1}{\Gamma\left( \alpha\right)\theta^{\alpha}}x^{\alpha-1}e^{-\frac{x}{\theta}}\frac{x}{N}\log\frac{x}{N}dx=\frac{S\alpha\theta}{N}\left( \log\frac{N}{\theta}-\psi\left( \alpha+1 \right) \right)$$

where $\psi(z)$ is the digamma function. Again in the same asymptotic limit, average abundance approaches its expected value from Eq. (A3.2), and we have

$$E\left( \log{}^{1}D \right)\sim\log(S\alpha)-\psi\left( \alpha+1 \right)$$

which, as for the lognormal, depends on only one of the distribution parameters ($\alpha$).

Then, for the gamma SAD, Pielou’s index for large $S$ is

$$E\left( J \right)=\frac{E(\log{}^{1}D)}{\log S}\sim\frac{1}{\log S}\left( \log(S\alpha)-\psi\left( \alpha+1 \right) \right)$$

(Again for large $S$, this approaches unity.)

And for large $S$ the Hill index is

$${}^{1}E=\frac{{}^{1}D}{S}\to\frac{1}{S}\exp\left( \log(S\alpha)-\psi\left( \alpha+1 \right) \right)\sim\alpha\exp\left( -\psi\left( \alpha+1 \right) \right)$$

which is independent of $\theta$ and $S$.

**Appendix S2: The Lorenz-consistency axioms and addition of rare species**

Let $\mathbf{n}$ be a vector of abundances (or incomes) for a set of individuals. The units of abundance are not specified, and the values should be interpreted only in relative terms because we will allow non-integer abundances. Let $E(\mathbf{n})$ be a scalar metric. The function $E(\mathbf{n})$ is a Lorenz-consistent evenness index (or a Lorenz-consistent equality index) if it satisfies four axioms (Foster 1985):

(i) Symmetry (anonymity): if $\mathbf{n}^{*}$ is a reordering of the elements of $\mathbf{n}$ then $E\left( \mathbf{n}^{*} \right)=E(\mathbf{n})$.
(ii) Replication: if $\mathbf{n}^{*}$ comprises the vector $\mathbf{n}$ repeated $k\in\mathbb{Z}^{+}$ times, then $E\left( \mathbf{n}^{*} \right)=E(\mathbf{n})$.
(iii) Scale invariance: if $K\in\mathbb{R}^{+}$, then $E\left( K\mathbf{n} \right)=E\left( \mathbf{n} \right).$
(iv) The Pigou–Dalton transfer principle: if $\mathbf{n}^{*}$ is obtained from $\mathbf{n}$ via a progressive transfer, then $E\left( \mathbf{n}^{*} \right)>E\left( \mathbf{n} \right)$. A progressive transfer is one that reduces the abundance (wealth) gap between two individuals; a regressive transfer is one that increases the gap.

*Conjecture*

If we have an abundance vector $\mathbf{n}$ of length $S>0$, comprising only positive abundances (i.e., absent species, with $n_{i}=0$, are not included), then for an evenness index that satisfies the Lorenz-consistency axioms the addition of a new sufficiently rare species will decrease evenness (increase inequality). Mathematically, if we call the vector with the new rare species added $\mathbf{n}^{*}$, then the conjecture is that $E\left( \mathbf{n} \right)>E(\mathbf{n}^{*})$.

*The case* $S=1$

For the case $S=1$, we have

$$\mathbf{n}=\{n_{1}\}$$

By the replication axiom we have

$$\begin{aligned} E\left( \mathbf{n} \right)=E\left( \left\{ n_{1} \right\} \right)=E\left( \left\{ n_{1},n_{1} \right\} \right)\#\left( A1.1 \right) \end{aligned}$$

Let $\kappa<n_{1}$ be some small positive number and $\delta=\frac{n_{1}\left( n_{1}-\kappa\right)}{n_{1}+\kappa}<n_{1}$. By the Pigou–Dalton transfer principle we have

$$\begin{aligned} E\left( \left\{ n_{1},n_{1} \right\} \right)>E\left( \left\{ n_{1}-\delta,n_{1}+\delta\right\} \right)\#\left( A1.2 \right) \end{aligned}$$

Then by the scale invariance axiom we have

$$\begin{aligned} E\left( \left\{ n_{1}-\delta,n_{1}+\delta\right\} \right) =E\left( \left\{ n_{1}-\delta,n_{1}+\delta\right\} \right) \times\frac{n_{1}}{n_{1}+\delta}=E\left( \left\{ \frac{n_{1}-\delta}{n_{1}+\delta}n_{1},n_{1} \right\} \right)=E\left( \left\{ \kappa,n_{1} \right\} \right)=E(\mathbf{n}^{*})\#\left( 3 \right) \end{aligned}$$

Putting Eqs. (1), (2) and (3) together we have the desired result

$$E\left( \mathbf{n} \right)=E\left( \left\{ n_{1} \right\} \right)>E\left( \left\{ \kappa,n_{1} \right\} \right)=E(\mathbf{n}^{*})$$

*The case* $S>1$

Now consider the general case $S>1$. Let $J=\sum_{i=1}^{S} n_{S}\geq S$ be the total abundance (e.g., if $n_{S}$ is measured in number of individual, $J$ is the community size). Note we are guaranteed $J>n_{1}$. We assume that the elements of $\mathbf{n}$ are sorted from smallest to largest (if a vector is not in this form, we can put it is in this form via the symmetry axiom without changing evenness).

The general approach to proving the conjecture for $S>1$ will be similar to that for the case $S=1$ above, but we need an extra application of the replication axiom and the required Pigou–Dalton transfers are more complicated.

First create a vector $\mathbf{n'}$ by replicating each element of the vector $\mathbf{n}$ a total of $S+1$ times (maintaining the ordering of the vector). Consider also a vector $\mathbf{n''}$, which is a vector of $S$ instances of some small number $\epsilon$ followed by $S$ instances of each element of $\mathbf{n}$, scaled such that the sum of all elements of $\mathbf{n''}$ is the same as that of $\mathbf{n'}$. Both of these vectors will have length $S(S+1)$. Specifically, the vector $\mathbf{n'}$ is

$$\mathbf{n}^{\mathbf{'}}=\{n_{1},n_{1},\ldots n_{1},n_{2},n_{2},\ldots n_{2},\ldots, n_{S},n_{S},\ldots n_{S}\}$$

where each element is replicated $S+1$ times, and the vector $\mathbf{n''}$ is

$$\mathbf{n}^{\mathbf{''}}=\left\{ \epsilon, \epsilon,\ldots\epsilon,n_{1},n_{1},\ldots n_{1},n_{2},n_{2},\ldots n_{2},\ldots, n_{S},n_{S},\ldots n_{S} \right\}\times K$$

where each element is replicated $S$ times, and $K$ is the scaling factor that guarantees that $\sum_{i=1}^{S(S+1)} n_{i}^{'}=\sum_{i=1}^{S(S+1)} n_{i}^{''}$. Specifically, we have

$$K=\frac{\left( S+1 \right)\sum_{i=1}^{S} n_{i}}{S\epsilon+S\sum_{i=1}^{S} n_{i}}=\frac{1+\frac{1}{S}}{\frac{\epsilon}{J}+1}$$

where $J=\sum_{i=1}^{S} n_{i}$.

We are allowed to assume arbitrarily small $\epsilon$, and in particular we will assume throughout that

$$\begin{aligned} \epsilon<\frac{n_{1}}{S}\equiv\epsilon^{*}\#\left( A1.3 \right) \end{aligned}$$

Note that Eq. (A1.3) guarantees that $K>1$ because

$$\epsilon<\frac{n_{1}}{S}\leq\frac{n_{s}}{S}<\frac{J}{S}$$

We also have $K<1+1/S$. Note also that the first $S$ elements of the vector $\mathbf{n}''$ are equal to

$$\kappa\equiv K\epsilon=\epsilon\frac{1+\frac{1}{S}}{\frac{\epsilon}{J}+1}$$

which is an increasing function of $\epsilon$.

We want to first show that for sufficiently small $\epsilon$, we can transform $\mathbf{n'}$ into $\mathbf{n''}$ via regressive transfers, i.e., transfers from donor elements with lower abundance to recipient elements with higher abundance. Note that any initial transfer from element $i$ to element $j$ will be regressive if $i<j$, because the vector is assumed to be sorted by increasing abundance; even if $n_{i}^{'}=n_{j}'$, the transfer will be regressive. Any subsequent transfer from element $i$ to element $j$ with $i<j$ will also be regressive, provided that element $i$ has not been the recipient of any previous transfers and element $j$ has not been a donor for any previous transfers. Let us enact the transfers using the following algorithm, which guarantees that any given element can only ever be a donor or a recipient:

(i) Create a “transfer pot” initially of size $X_{0}=0$.
(ii) Set $i=1$.
(iii) If element $i$ of $\mathbf{n'}'$ is equal to element $i$ of $\mathbf{n}'$, then this is neither a donor nor a recipient element, so set $X_{i}=X_{i-1}$ and go to step (vi).
(iv) If element $i$ of $\mathbf{n'}'$ is less than element $i$ of $\mathbf{n}'$, then this is a donor element, so add $\Delta n_{i}=n_{i}^{'}-n_{i}^{''}$ to the transfer pot, giving $X_{i}=X_{i-1}+\Delta n_{i}$. Then go to step (vi).
(v) If element $i$ of $\mathbf{n'}'$ is greater than element $i$ of $\mathbf{n}'$, then this is a recipient element, so take $-\Delta n_{i}=-\left( n_{i}^{'}-n_{i}^{''} \right)$ from the transfer pot, giving $X_{i}=X_{i-1}+\Delta n_{i}$. This step will constitute a valid regressive transfer as long as our pot is not empty, i.e., $X_{i}>0$, which is equivalent to $X_{i-1}>-\Delta n_{i}=\left| \Delta n_{i} \right|$ (see below).
(vi) If $i=S$ then end.
(vii) Increment $i$ and go to step (iii).

The key to proving that all the transfers in the above algorithm are valid regressive transfers under the Pigou–Dalton principle is to show that we always have non-negative cumulative partial sums, $X_{k}\geq0$, for the vector $\mathbf{n}^{\mathbf{'}}-\mathbf{n''}$ (this implies that the pot is always big enough for any required transfer, without the pot going into debt):

$$X_{k}=\sum_{i=1}^{k} \left( n_{i}^{'}-n_{i}^{''} \right)\geq0$$

Let $i=(a-1)\left( S+1 \right)+b$, where $a\in\{1,2,\ldots S\}$ and $b\in\{1,2,\ldots S+1\}$. We have $n_{i}^{'}=n_{a}$. For $a=1$, we have

$$n_{i}^{''}=\left\{ \begin{matrix} K\epsilon, & b\leq S \\ Kn_{1}, & b=S+1 \end{matrix} \right.$$

and for $a>1$, we have

$$n_{i}^{''}=\left\{ \begin{matrix} Kn_{a-1}, & b\leq S+1-a \\ Kn_{a}, & b>S+1-a \end{matrix} \right.$$

Note that for the special case $k=S(S+1)$, we have $X_{k}=0$ by design (the vectors $\mathbf{n}'$ and $\mathbf{n}''$ have the same sums, enforced by the factor $K$).

Now consider the general case $k<S(S+1)$. We can rewrite the partial sums as

$$X_{k}=\sum_{a=1}^{A-1} \sum_{b=1}^{S+1} (n_{(a-1)\left( S+1 \right)+b}^{'}-n_{(a-1)\left( S+1 \right)+b}^{''})+\sum_{b=1}^{B} (n_{(A-1)\left( S+1 \right)+b}^{'}-n_{(A-1)\left( S+1 \right)+b}^{''})$$

where

$$B=1+\left( k-1 \right)\mathrm{mod} \left( S+1 \right)$$

$$A=\left\lceil\frac{k}{S+1} \right\rceil$$

for $k=\left( A-1 \right)\left( S+1 \right)+B\in\left\{ 1,2,\ldots S\left( S+1 \right) \right\}$. We will break the proof into four sub-cases:

(i) $A=1$, $B\leq S$
(ii) $A=1, B=S+1$
(iii) $A>1$, $B\leq S+1-A$
(iv) $A>1, B>S+1-A$

For sub-cases with $A=1$, we have $k=B$ and thus

$$\begin{aligned} X_{k}=\sum_{b=1}^{B} \left( n_{b}^{'}-n_{b}^{''} \right)=\sum_{b=1}^{B} \left( n_{1}-n_{b}^{''} \right)=Bn_{1}-\min\left( S,B \right)K\epsilon-\max\left( B-S,0 \right)Kn_{1}\#\left( A1.4 \right) \end{aligned}$$

*Sub-case (i):* $A=1$*,* $B\leq S$

For $B\leq S$, Eq. (A1.4) becomes $X_{k}=B\left( n_{1}-K\epsilon\right)$, which is non-negative if $n_{1}-K\epsilon\geq0$, which is true if

$$\begin{aligned} \epsilon\leq\frac{n_{1}S}{\left( 1-\frac{n_{1}}{J} \right)S+1}\#\left( A1.5 \right) \end{aligned}$$

But from Eq. (A1.3), we have

$$\epsilon<\frac{n_{1}}{S}<\frac{n_{1}S}{\left( 1-\frac{n_{1}}{J} \right)S+1}$$

(which can be proven by multiplying out the denominators and simplifying, leading to $\left( 1-\frac{n_{1}}{J} \right)S+1<S^{2}$, which is true because $\left( 1-\frac{n_{1}}{J} \right)S+1<S+1<S^{2}$, for $S\geq2$), and so Eq. (A1.5) holds and thus $X_{k}$ is non-negative for this sub-case.

*Sub-case (ii):* $A=1$*,* $B=S+1$

For $B=S+1$, Eq. (A1.4) becomes $X_{k}=Bn_{1}-SK\epsilon-\left( B-S \right)Kn_{1}=(S+1-K)n_{1}-SK\epsilon$, which is positive if we assume

$$\begin{aligned} \epsilon\leq\frac{n_{1}\left( S-1 \right)}{\left( 1-\frac{n_{1}}{J} \right)S}\#\left( A1.6 \right) \end{aligned}$$

But from Eq. (A1.3), we have

$$\epsilon<\frac{n_{1}}{S}<\frac{n_{1}\left( S-1 \right)}{\left( 1-\frac{n_{1}}{J} \right)S}$$

(which is easy to see because the inequality just becomes $2<S+\frac{n_{1}}{J}$, which must hold because $S\geq2$), and so Eq. (A1.6) holds and $X_{k}$ is non-negative for this sub-case.

*Sub-case (iii):* $A>1$*,* $B\leq S+1-A$

If $A>1$, we can simplify $X_{k}$ as follows:

$$X_{k}=\sum_{a=1}^{A-1} \sum_{b=1}^{S+1} \left( n_{a}-n_{\left( a-1 \right)\left( S+1 \right)+b}^{''} \right)+\sum_{b=1}^{B} \left( n_{A}-n_{\left( A-1 \right)\left( S+1 \right)+b}^{''} \right)=\left( S+1 \right)n_{1}-SK\epsilon-Kn_{1}+\sum_{a=2}^{A-1} \left\{ \left( S+1 \right)n_{a}-\left( S+1-a \right)Kn_{a-1}-aKn_{a} \right\}+Bn_{A}-\min\left( S+1-A,B \right)Kn_{A-1}-\max\left( B-\left( S+1-A \right),0 \right)Kn_{A}$$

$$\begin{aligned} \#\left( A1.7 \right) \end{aligned}$$

The term with the summation is

$$\sum_{a=2}^{A-1} \left\{ \left( S+1 \right)n_{a}-\left( S+1-a \right)Kn_{a-1}-aKn_{a} \right\}=\sum_{a=2}^{A-1} \left( S+1-aK \right)n_{a}-\sum_{a=1}^{A-2} \left( S-a \right)Kn_{a}=\sum_{a=2}^{A-2} \left( 1-S\left( K-1 \right) \right)n_{a}+\left( S+1-\left( A-1 \right)K \right)n_{A-1}-\left( S-1 \right)Kn_{1}$$

Assuming $B\leq S+1-A$, the last three terms in the expression for $X_{k}$ in Eq. (A1.7) become

$$Bn_{A}-BKn_{A-1}=B\left( n_{A}-Kn_{A-1} \right)$$

We thus have

$$X_{k}=\left( S+1 \right)n_{1}-SK\epsilon-Kn_{1}+\sum_{a=2}^{A-2} \left( S\left( 1-K \right)+1 \right)n_{a}+\left( S+1-\left( A-1 \right)K \right)n_{A-1}-\left( S-1 \right)Kn_{1}+B\left( n_{A}-Kn_{A-1} \right)=\left( 1-S(K-1) \right)\sum_{a=1}^{A-1} n_{a}+\left( S+1-A \right)Kn_{A-1}+Bn_{A}-BKn_{A-1}-SK\epsilon$$

We want to show that

$$\left( 1-S\left( K-1 \right) \right)\sum_{a=1}^{A-1} n_{a}+\left( S+1-A \right)Kn_{A-1}+Bn_{A}-BKn_{A-1}-SK\epsilon\geq0$$

$$\begin{aligned} \Rightarrow\left( 1-S\left( K-1 \right) \right)\sum_{a=1}^{A-1} n_{a}+\left( S+1-A \right)Kn_{A-1}+Bn_{A}\geq BKn_{A-1}+SK\epsilon\#\left( A1.8 \right) \end{aligned}$$

Define $J_{A-1}=\sum_{a=1}^{A-1} n_{a}.$ We have

$$\begin{aligned} \frac{J-J_{A-1}}{n_{A}}=\frac{1}{n_{A}}\sum_{a=A}^{S} n_{a}=1+\frac{n_{A+1}}{n_{A}}+\ldots+\frac{n_{S}}{n_{A}}\#\left( A1.9 \right) \end{aligned}$$

which must be decreasing with $A$, because if we increase $A$ we reduce the number of terms and we reduce the size of each term. The maximum value of Eq. (A1.9) thus arises for $A=1$ and the minimum value for $A=S$:

$$\begin{aligned} 1\leq\frac{J-J_{S-1}}{n_{S}}\leq\frac{J-J_{A-1}}{n_{A}}\leq\frac{J-J_{0}}{n_{1}}=\frac{J}{n_{1}}\#\left( A1.10 \right) \end{aligned}$$

Going back to Eq. (A1.8) and rearranging gives

$$\frac{1+\frac{1}{S}}{\frac{\epsilon}{J}+1}\left\{ -SJ_{A-1}+\left( S+1-A \right)n_{A-1}-Bn_{A-1} \right\}+\left( S+1 \right)J_{A-1}+Bn_{A}\geq S\frac{1+\frac{1}{S}}{\frac{\epsilon}{J}+1}\epsilon$$

$$\Rightarrow-SJ_{A-1}+\left( S+1-A \right)n_{A-1}-Bn_{A-1}+\left( SJ_{A-1}+\frac{BSn_{A}}{S+1} \right)\left( \frac{\epsilon}{J}+1 \right)\geq S\epsilon$$

$$\Rightarrow J\left( Bn_{A}S+\left( S+1 \right)\left( S+1-A-B \right)n_{A-1} \right)\geq\epsilon S\left( \left( S+1 \right)\left( J-J_{A-1} \right)-Bn_{A} \right)$$

The left-hand side is positive because $S+1-A-B\geq0$. The right-hand side is positive because $S+1>B$ (from $S+1-A\geq B$) and $J-J_{A-1}\geq n_{A}$. So we have

$$\begin{aligned} \epsilon\leq\frac{J\left( BSn_{A}+\left( S+1 \right)\left( S+1-A-B \right)n_{A-1} \right)}{S\left( \left( S+1 \right)\left( J-J_{A-1} \right)-Bn_{A} \right)}\#\left( A1.11 \right) \end{aligned}$$

To show that the expression on the right-hand side of Eq. (A1.11) is greater than $\epsilon^{*}=n_{1}/S$ (Eq. (A1.3)), we do some algebra to obtain the following desired inequality:

$$BS+\left( S+1 \right)\left( S+1-A-B \right)\frac{n_{A-1}}{n_{A}}+\frac{n_{1}}{J}B>\frac{n_{1}}{J}\left( S+1 \right)\frac{J-J_{A-1}}{n_{A}}$$

We know from Eq. (A1.10) that

$$\frac{J-J_{A-1}}{n_{A}}\leq\frac{J}{n_{1}}$$

and so

$$\frac{n_{1}}{J}\left( S+1 \right)\frac{J-J_{A-1}}{n_{A}}\leq S+1$$

from which the desired inequality follows for $B>1$ because $BS\geq2S\geq S+1$. To show that the right-hand side of Eq. (A1.11) is greater than $n_{1}/S$ also for $B=1$, we again do some algebra, starting from the desired condition:

$$J\left( S+\left( S+1 \right)\left( S-A \right)\frac{n_{A-1}}{n_{A}} \right)\geq n_{1}\left( \left( S+1 \right)\frac{J-J_{A-1}}{n_{A}}-1 \right)$$

$$\Rightarrow J\left( Sn_{A}+\left( S+1 \right)\left( S-A \right)n_{A-1} \right)+n_{1}\left( \left( S+1 \right)\left( n_{1}+n_{2}+\ldots+n_{A-1} \right)+n_{A} \right)\geq\left( S+1 \right)Jn_{1}$$

The left-hand side here is positive because $S\geq A$, and on the left-hand side we have a term $\left( S+1 \right)\left( S-A \right)Jn_{A-1}$, which is greater than the right-hand side, $\left( S+1 \right)Jn_{1}$, provided that $S\neq A$, and so the inequality holds. For the case $S=A$, the desired condition becomes

$$JS\geq n_{1}\left( \left( S+1 \right)\frac{n_{S}}{n_{S}}-1 \right)$$

$$\Rightarrow JS\geq n_{1}S$$

$$\Rightarrow J\geq n_{1}$$

which is true. And so Eq. (A1.11) holds for this sub-case, implying $X_{k}\geq0$.

*Sub-case (iv):* $A>1$*,* $B>S+1-A$

If $B>S+1-A$ then the last three terms in the expression for $X_{k}$ in Eq. (A1.7) become

$$Bn_{A}-\left( S+1-A \right)Kn_{A-1}-\left( B-\left( S+1-A \right) \right)Kn_{A}$$

We thus have

$$X_{k}=\left( S+1 \right)n_{1}-SK\epsilon-Kn_{1}+\sum_{a=2}^{A-2} \left( 1-S\left( K-1 \right) \right)n_{a}+\left( S+1-\left( A-1 \right)K \right)n_{A-1}-\left( S-1 \right)Kn_{1}+Bn_{A}-\left( S+1-A \right)Kn_{A-1}-\left( B-\left( S+1-A \right) \right)Kn_{A}=\left( 1-S\left( K-1 \right) \right)n_{1}-SK\epsilon+\sum_{a=2}^{A-2} \left( 1-S\left( K-1 \right) \right)n_{a}+\left( 1-S\left( K-1 \right) \right)n_{A-1}+\left\{ B-\left( B-\left( S+1-A \right) \right)K \right\}n_{A}=\left( 1-S(K-1) \right)\sum_{a=1}^{A-1} n_{a}+\left( S+1-A \right)Kn_{A}+Bn_{A}-BKn_{A}-SK\epsilon$$

We want to show that

$$\left( 1-S(K-1) \right)\sum_{a=1}^{A-1} n_{a}+\left( S+1-A \right)Kn_{A}+Bn_{A}-BKn_{A}-SK\epsilon\geq0$$

$$\Rightarrow\left( 1-S(K-1) \right)\sum_{a=1}^{A-1} n_{a}+\left( S+1-A \right)Kn_{A}\geq B\left( K-1 \right)n_{A}+SK\epsilon$$

Following similar logic to that leading to Eq. (A1.11) (just with $n_{A}$ in place of $n_{A-1}$), we end up with the desired inequality

$$Jn_{A}\left( BS+\left( S+1 \right)\left( S+1-A-B \right) \right)\geq\epsilon S\left( \left( S+1 \right)\left( J-J_{A-1} \right)-Bn_{A} \right)$$

The left-hand side is non-negative, reaching its lowest value of zero when $A=S$ and $B=S+1$, a special case that we handled earlier (the case $k=S(S+1))$. The right-hand side is non-negative, reaching zero only in the special case $S+1=B$ and $J-J_{A-1}=n_{A}$, in which case the inequality is trivially satisfied. Otherwise, we have the desired inequality

$$\begin{aligned} \epsilon\leq\frac{Jn_{A}\left( BS+\left( S+1 \right)\left( S+1-A-B \right) \right)}{S\left( \left( S+1 \right)\left( J-J_{A-1} \right)-Bn_{A} \right)}=\frac{J\left( \left( S+1 \right)\left( S+1-A \right)-B \right)}{S\left( \left( S+1 \right)\frac{J-J_{A-1}}{n_{A}}-B \right)}\#\left( A1.12 \right) \end{aligned}$$

To prove this, we want to use Eq. (A1.3) ($\epsilon<\epsilon^{*})$ to show that

$$\epsilon<\epsilon^{*}=\frac{n_{1}}{S}\leq\frac{J\left( \left( S+1 \right)\left( S+1-A \right)-B \right)}{S\left( \left( S+1 \right)\frac{J-J_{A-1}}{n_{A}}-B \right)}$$

For $A=S$, the inequality reduces to $n_{1}\leq J$, which is true. For $A<S$, note first that the right-hand side of Eq. (A1.12) is a decreasing function of $B$. We know this because

$$S-A<\frac{n_{A+1}}{n_{A}}+\ldots+\frac{n_{S}}{n_{A}}$$

(the right-hand side sums over $S-A$ values, all of which are greater than or equal to one) and thus

$$S+1-A<\frac{J-J_{A-1}}{n_{A}}$$

The minimum value of the right-hand side of Eq. (A1.12) thus occurs when $B=S+1$, in which case Eq. (A1.12) becomes

$$\epsilon\leq\frac{J\left( S-A \right)}{S\left( \frac{J-J_{A-1}}{n_{A}}-1 \right)}$$

And note that

$$\frac{J\left( S-A \right)}{S\left( \frac{J-J_{A-1}}{n_{A}}-1 \right)}\geq\frac{J\left( S-\left( S-1 \right) \right)}{S\left( \frac{J}{n_{1}}-1 \right)}>\frac{J}{S\left( \frac{J}{n_{1}} \right)}=\frac{n_{1}}{S}$$

which is the required inequality. Thus Eq. (A1.12) holds for this sub-case and $X_{k}>0$.

(Note that in the above proof we showed that the vector $\mathbf{n}'$ can be converted into the vector $\mathbf{n}''$ using Pigou–Dalton transfers provided that $\epsilon<\epsilon^{*}=n_{1}/S$. For the given sequence of transfers, there is no higher value of $\epsilon^{*}$ that works for arbitrary communities because, for example, if we have the community $\{1,1,n_{3}\}$, then in the limit of large $n_{3}$ the right-hand side of Eq. (A1.12) approaches $\frac{1}{3}$ for $A=2$ and $B=4$. Graphical investigations with Lorenz curves suggest that $\epsilon^{*}=n_{1}/S$ is in fact a hard limit for the conjecture to be true for arbitrary species abundance distributions (SADs) given values of $n_{1}$ and $S$, and that it is not just a constraint of this particular proof. This also shows that there is no trivial proof of the conjecture because the proof necessarily involves identification of the threshold $\epsilon^{*}=n_{1}/S$, which apparently does not have a simple intuitive basis.)

*Final steps*

We can now prove the conjecture as follows:

(i) Via the replication axiom we have $E\left( \mathbf{n} \right)=E(\mathbf{n}^{'})$.
(ii) Via the Pigou–Dalton transfer principle we have $E\left( \mathbf{n}^{\mathbf{'}} \right)>E(\mathbf{n}^{''})$.
(iii) Via the scaling invariance axiom we have $E\left( \mathbf{n}^{''} \right)=E\left( \mathbf{n}^{''}/K \right)$.
(iv) Via the replication axiom (applied in reverse to create the vector $\mathbf{n}^{*}$, which has length $S+1$, from the vector $\mathbf{n}^{''}/K$, which has length $S(S+1)$), we have $E(\mathbf{n}^{''}/K)=E(\mathbf{n}^{*})$.
(v) Putting the above steps together we see that $E\left( \mathbf{n} \right)>E(\mathbf{n}^{*})$, i.e., $E\left( \left\{ n_{1},n_{2},\ldots n_{S} \right\} \right)>E\left( \left\{ \epsilon,n_{1},n_{2},\ldots n_{S} \right\} \right)$, which means that adding a rare species decreases evenness (increases inequality).

*Numerical example*

For example, if we have the abundance vector $\mathbf{n}=\{1,2\}$, then $\mathbf{n'}=\{1,1,1,2,2,2\}$ and $\mathbf{n}^{''}=\left\{ \epsilon,\epsilon,1,1,2,2 \right\}\times K$, where $K=\frac{9}{6+2\epsilon}$. Let $\epsilon=0.1<\frac{n_{1}}{S}=\frac{1}{3}$, so that $\mathbf{n}^{''}=\left\{ 0.145, 0.145, 1.452, 1.452, 2.903, 2.903 \right\}$. and we can obtain $\mathbf{n}^{''}$ from $\mathbf{n'}$ via the following regressive transfers:

- Transfer $0.452$ units from element 1 to element 3;
- Transfer $0.403$ units from element 1 to element 5;
- Transfer $0.500$ units from element 2 to element 5;
- Transfer $0.355$ units from element 2 to element 6;
- Transfer $0.548$ units from element 4 to element 6.

Then we scale the vector $\mathbf{n}''$ by $1/K$ to get the vector $\{0.1, 0.1, 1, 1, 2, 2\}$, which has the same evenness (via the scale invariance axiom), and lastly we use the replication axiom to get the vector $\mathbf{n}^{*}=\{0.1, 1, 2\}$, which again has the same evenness.

**Appendix S3: The Lorenz-consistency axioms and Jost’s evenness property**

Jost (2010) proposed an alternative axiomatisation of the evenness problem, which involves the property that the range of an evenness index not be constrained by the value of species richness (he calls this property “independence”, but I avoid this terminology here for reasons discussed in the main text). Here I show that the Jost’s property is incompatible with the Lorenz-consistency axioms (see Appendix S2).

Suppose we have an evenness metric $V$ that satisfies Jost’s property. Call $V_{0}$ the minimum attainable value of $V$, and let $J$ be the community size. Then, for a two-species community we have

$$\lim_{\epsilon\to0} V\left( \left\{ J-\epsilon,\epsilon\right\} \right)=V_{0}$$

Replication invariance (Lorenz-consistency axiom (ii)) requires that

$$\lim_{\epsilon\to0} V\left( \left\{ J-\epsilon,\epsilon,J-\epsilon,\epsilon\right\} \right)=V_{0}$$

But by Jost’s property, we also have

$$\lim_{\epsilon\to0} V\left( \left\{ 2J-3\epsilon,\epsilon,\epsilon,\epsilon\right\} \right)=V_{0}$$

But this contradicts the transfer principle (Lorenz-consistency axiom (iv)), whereby we should have

$$V\left( \left\{ 2J-3\epsilon,\epsilon,\epsilon,\epsilon\right\} \right)<V\left( \left\{ J-\epsilon,\epsilon,J-\epsilon,\epsilon\right\} \right)$$

for all $\epsilon$.

**Appendix S4: Evenness and biomass**

In this appendix, I describe the methods used in the first case study in the main text, which explores the relationship between evenness and biomass in a consumer–resource model. The consumer–resource model comprises $n$ species competing for $m$ resources. The dynamics are governed by the following equations (Tilman 1981):

$$\begin{aligned} \frac{dN_{i}}{dt}=N_{i}g_{i}\left( R_{1},R_{2},\ldots R_{m} \right)-DN_{i}\#\left( 13a \right) \end{aligned}$$

$$\begin{aligned} \frac{dR_{j}}{dt}=DS_{j}-DR_{j}-\sum_{i=1}^{n} Q_{ij}N_{i}g_{i}\left( R_{1},R_{2},\ldots R_{m} \right)\#\left( 13b \right) \end{aligned}$$

where

$$g_{i}\left( R_{1},R_{2},\ldots R_{m} \right)=\min_{j\in\left\{ 1,2,\ldots m \right\}} \left\{ \frac{r_{ij}R_{j}}{h_{ij}+R_{j}} \right\}$$

and $t$ is time (units of days, d), $N_{i}$ is the density of species $i$ (cell/L), $R_{j}$ is the concentration of resource $j$ ($\mu$mol/L), $r_{ij}$ is the maximum growth rate of species $i$ when limited by resource $j$ (d^-1^), $h_{ij}$ is the half-saturation constant of species $i$ consuming resource $j$ ($\mu$mol/L), $D$ is the fractional inflow rate of new medium and outflow rate of old medium (d^-1^), $S_{j}$ is the concentration of resource $j$ in the input medium ($\mu$mol/L), and $Q_{ij}$ is the amount of resource $j$ required to make one unit of species $i$ ($\mu$mol/cell). Units are omitted henceforth for brevity.

I parameterised the model for $n=4$ species and $m=4$ resources in a manner that allows each species $i$ to specialise on the corresponding resource $i$ and gives a high probability of four-species coexistence:

- The flow rate parameter was set to $D=1$, and the resource input concentrations were all set to $S_{j}=10$.
- The half-saturation constants for each species $i$ on the corresponding resource $i$ were set to $h_{ii}=1$. The other $h_{ij}$ parameters were drawn randomly from the interval $[0, 0.1]$.
- The resource-conversion parameters for each species $i$ on the corresponding resource $i$ were set to $Q_{ii}=0.2$. The other resource-conversion parameters were all set to $Q_{ij}=1$.
- The $r_{ij}$ parameters were drawn randomly from the interval $D\times[1.1, 2.1]$.
- The initial resource concentrations were set to the input concentrations, $R_{j}=S_{j}$.
- The initial species densities $N_{i}$ were drawn randomly from the interval $[0, 10]$.

I ran each simulation for a duration of $10,000$ time units, which was sufficient to reach equilibrium. Limit cycles and chaotic dynamics are possible for this model (Huisman & Weissing 1999) but were not observed for my parameterisations. At the end of each simulation I calculated biomass by summing all of the species’ densities—this effectively assumes that each species has the same per-cell biomass, but results would be robust to relaxation of this assumption. I also calculated Hill evenness ${}^{1}E$. I ran 100 repeats of the model (each time redrawing the parameters from the uniform distributions where appropriate) and explored the relationship between total biomass and evenness (Fig. 2).

**Appendix S5: Immigration and evenness**

In this appendix, I describe the methods for the second case study in the main text, which explores the relationship between immigration and evenness in a theoretical model. The model is the spatially implicit neutral model (Hubbell 2001; Volkov *et al.* 2003).

The model comprises a local community and a metacommunity. The metacommunity comprises $J_{M}$ individuals. At each timestep, an individual is chosen to die at random and is replaced by the offspring of a randomly chosen parent. With probability $\nu$ (typically small), this offspring mutates to a new species. The expected SAD in the metacommunity is approximately log-series (the approximation becomes exact in the limit of high diversity; Chisholm & Pacala 2010). The local community comprises $J$ individuals. At each timestep an individual is chosen to die at random and is replaced by the offspring of a randomly chosen parent within the local community or the metacommunity. The former occurs with probability $1-m$ and the latter with probability $m$, and so $m$ is conceptualised as the per-capita immigration probability. The metacommunity is typically treated as static on timescales relevant to the local community. The diversity of the local community is thus wholly determined by $J$, $m$, and the SAD in the metacommunity.

I generated local communities with expected size $J$ and expected species richness $S$ using the fisher.ecosystem() function in the untb R package. The fisher.ecosystem() is designed to generate communities for metacommunities, not local communities. To adapt it to my purpose, I used the following algorithm to generate a single realisation of a local community for given values of $J$, expected local community richness $S$, and $m$:

(1) I conceptualised the local community as a community of lineages, each arising from a single immigration event. This makes the model mathematically equivalent to a metacommunity, with speciation being equal to the immigration rate ($\nu=m$). I used the fisher.ecosystem() function to generate a lineage identity for each individual in the local community.
(2) I created a metacommunity with size $J_{M}={10}^{8}$ and speciation rate $\nu=\theta/(\theta+J_{M}-1)$, where $\theta=S/(q-W_{-1}\left( qe^{q} \right))$, and $q=S(1-m)/(Jm\log m)$ (the function $W_{-1}\left( z \right)$ is the lower branch of the Lambert W function). This parameterisation ensures that the expected number of species in the local community will be $S$, as required. (This can be verified from formulas in the supplementary material of Chisholm *et al.* (2016).)
(3) For the $L$ distinct lineages arising in step (1), each of which arose from a distinct immigration event, I sampled a random individual from the metacommunity created in step (2). Some of the lineages will represent the same species (i.e., the same species immigrated more than once), and so we have $S\leq L$, with typically $S<L$.

I used a local community size of $J={10}^{6}$ and I set expected local community richness to $S=900$. I used four focal immigration rates $m={10}^{-4}$, $m={10}^{-3}$, $m={10}^{-2}$, $m={10}^{-1}$, and in addition 50 evenly spaced values on a logarithmic scale ranging from

$$m=-\frac{S}{J W_{-1}\left( -\frac{S}{J}\exp\left( -\frac{S}{J} \right) \right)}$$

which is the minimum immigration rate able to give expected species richness $S$ given expected size $J$, up to $m=0.9$. I generated 1000 independent communities for each value of $m$ and calculated the SAD and Hill evenness ${}^{1}E$ of each community. The resulting SADs for the four focal values of $m$ are shown in Fig. 3A. The resulting relationship of evenness to $m$ is shown in Fig. 3B.

**Appendix S6: Robustness of evenness indices to sampling**

To explore the robustness of evenness indices to sampling, I applied Hill evenness (${}^{1}E$) to synthetic datasets (similar results, not shown are obtained for one minus the Gini index, $1-G$). The datasets were generated from a variety of SADs. Let the number of species be $S$ and the expected number of individuals be $N$. The first SAD was (a stochastic sample of) a log-series distribution, whose single parameter is fully determined by $N$ and $S$. The second was a Poisson lognormal distribution with $\sigma=1$ and $\mu=\log\left( N/S \right)-\sigma^{2}/2$. The third was a uniform distribution in which all species had exactly $N/S$ individuals. The fourth was a bimodal distribution in which half the species each had just one individual, and the others each had $2S/N-1$ individuals. The first two SADs are intended to represent realistic scenarios; the latter two are deliberately chosen to be pathological to facilitate study of evenness indices under sampling in extreme situations.

For each distribution, I calculated the values of the evenness indices for sample sizes ranging from just one individual to all of the individuals in the dataset, where sampling is without replacement. This sampling regime aims to simulate a proportion of some individuals being missed in a field survey. I repeated the entire procedure 100 times, and computed mean evenness for each combination of distribution and sample size. I first ran the analysis for $N=1000$ and $S=10$, and then repeated it for $N=10,000$ and $S=100$.

Evenness tended to increase as the proportion of the community sampled decreased, with the exception of the uniform community (which has maximum evenness ${}^{1}E=1$ and so sampling can only decrease evenness). Evenness was somewhat robust to subsampling down to sample sizes of roughly 50%, but the bimodal distribution showed exceptionally low robustness, the reason being that sampling rapidly eliminates the many singleton species (Figs. S6.1–6.2). Variation in evenness across samples was greater for smaller communities (Fig. S6.1) than larger ones (Fig. S6.2), an expected result of the central limit theorem.

*Fig. S6.1.* Community evenness tends to be overestimated from samples, although estimates are somewhat robust down to a sample size of ≈50%, here with the exception of the case where the SAD is bimodal. Grey points show evenness estimates from individual samples and red curves show average evenness values for each sample size. Each panel is for a distinct SAD (panel titles). These simulations are for communities with $S=10$ species and an expected community size of $N=1,00$0.

*Fig. S6.2.* As for Fig. S6.1, but with $S=100$ and $N=10,000$.

*References*

Chisholm R.A., Fung T., Chimalakonda D. & O'Dwyer J.P. (2016). Maintenance of biodiversity on islands. *Proc. Roy. Soc. B*, 283, 20160102.

Chisholm R.A. & Pacala S.W. (2010). Niche and neutral models predict asymptotically equivalent species abundance distributions in high-diversity ecological communities. *Proc. Natl. Acad. Sci. USA*, 107, 15821–15825.

Foster J.E. (1985). Inequality measurement. In: *Fair Allocation* (ed. Young HP). American Mathematical Society Providence, RI, pp. 31–68.

Hubbell S.P. (2001). *The Unified Neutral Theory of Biodiversity and Biogeography*. Princeton University Press, Princeton, NJ.

Huisman J. & Weissing F.J. (1999). Biodiversity of plankton by species oscillations and chaos. *Nature*, 402, 407–410.

Jost L. (2010). The relation between evenness and diversity. *Diversity*, 2, 207–232.

May R.M. (1975). Patterns of Species Abundance and Diversity. In: *Ecology and Evolution of Communities* (eds. Cody ML & Diamond JM). Cambridge, Massachusetts, New England The Belknap Press of Harvard University Press, pp. 81–120.

Taillie C. (1979). Species Equitability: A Comparative Approach. In: *Ecology Diversity in Theory and Practice* (eds. Grassle JF, Patil GP, Smith W & Taillie C). International Co-operataive Publishing House Fairland, Maryland USA, pp. 51–61.

Tilman D. (1981). Tests of resource competition theory using four species of Lake Michigan algae. *Ecology*, 62, 802–815.

Volkov I., Banavar J.R., Hubbell S.P. & Maritan A. (2003). Neutral theory and relative species abundance in ecology. *Nature*, 424, 1035–1037.
